# Parasagittal subdural space: a novel quantitative marker of spontaneous intracranial hypotension syndrome-induced chronic subdural hematoma

**DOI:** 10.1186/s12880-025-02065-6

**Published:** 2025-12-29

**Authors:** Takahiro Tanaka, Hajime Takase, Tatsuya Haze, Wataru Shimohigoshi, Mitsuru Sato, Tetsuya Yamamoto

**Affiliations:** 1 Department of Neurosurgery, Yokohama City University, Yokohama, Kanagawa Japan; 2 Departments of Radiology and Neurology, Massachusetts General Hospital and Harvard Medical School, 149 13th St., Charlestown, MA 02129 USA; 3 YCU Center for Novel and Exploratory Clinical Trials (Y-NEXT), Yokohama City University Hospital, Yokohama, Japan; 4 Department of Medical Science and Cardiorenal Medicine, Yokohama City University Hospital, Graduate School of Medicine, Yokohama, Japan; 5 Department of Nephrology and Hypertension, Yokohama City University Medical Center, Yokohama, Japan

**Keywords:** Chronic subdural hematoma, Spontaneous intracranial hypotension, Cerebrospinal fluid, Computed tomography, Subdural space

## Abstract

**Background:**

Spontaneous intracranial hypotension syndrome (SIH)-induced chronic subdural hematoma (CSDH) often presents with orthostatic headaches but is frequently misdiagnosed, leading to inappropriate treatments like fatal hematoma drainage instead of epidural blood patches. In clinical practice, reliable and quantitative diagnostic criteria for this condition are lacking. This study uses initial CT scans to identify novel radiographic markers for accurately diagnosing SIH-induced CSDH.

**Methods:**

We retrospectively reviewed 310 consecutives hospitalized CSDH cases from January 2008 to May 2023. Among these, 54 were bilateral, with 11 induced by SIH; two secondary intracranial hypotension cases were excluded. We analyzed nine primary SIH-induced cases, comparing clinical and preoperative CT features with 43 non-SIH bilateral cases, focusing on the parasagittal subdural space (PSS) volume. We also conducted propensity score matching to validate our findings.

**Results:**

Patients with SIH-induced bilateral CSDH were significantly younger than those without SIH (mean age 54.7 vs. 76.2 years; *P* < 0.001). Orthostatic headache was more common in the SIH group (66.7% vs. 2.3%, *P* < 0.001). While hematoma volumes were similar, PSS volume was significantly larger in the SIH group (mean 15.0 vs. 5.1 mL, *P* = 0.007). ROC analysis identified an exploratory PSS cut-off of 11.1 mm², which yielded a sensitivity of 86% and a specificity of 66.7% (*P* = 0.009). Linear regression and qualitative assessments indicated a significant association between PSS volume and crural-and-ambient cistern obliteration, as well as cerebellar ptosis in the SIH group (*P* < 0.001).

**Conclusion:**

A preserved PSS on coronal CT represents a novel, quantitative marker for SIH-induced CSDH and may serve as a practical diagnostic clue, particularly when MRI is unavailable.

**Supplementary Information:**

The online version contains supplementary material available at 10.1186/s12880-025-02065-6.

## Introduction

Spontaneous intracranial hypotension (SIH) results from cerebrospinal fluid (CSF) leakage into the extradural space without any preceding trauma or medical manipulation [1–6]. SIH can be diagnosed according to the International Classification of Headache Disorders, primarily based on clinical symptoms and radiological evidence of CSF hypovolemia or leakage. However, despite increasing awareness and imaging advancements, SIH remains frequently underdiagnosed or misdiagnosed, even though several suggestive radiological features have been reported [7–9].

Chronic subdural hematoma (CSDH) is a common cause of headache and neurological symptoms, particularly in the elderly [10]. Most cases are considered trauma-related, and increasing evidence suggests that local immune cells and vascular permeability contribute to its pathogenesis [11, 12]. Notably, CSDH occurs in 16–57% of SIH cases and typically presents bilaterally due to brain sagging associated with intracranial hypotension (IH) and reduced CSF volume [13–19]. SIH-induced CSDH can result in disabling headaches or even coma, and while SIH is generally managed conservatively or with epidural blood patching (EBP), CSDH with mass effect often requires surgical drainage [5, 20–23]. Misdiagnosing SIH-induced CSDH as non-SIH-related can lead to unnecessary surgery, recurrence and potentially serious outcome, whereas delayed evacuation in cases of mass effect may cause neurological deterioration [3, 14, 22–26].

**Fig. 1:**
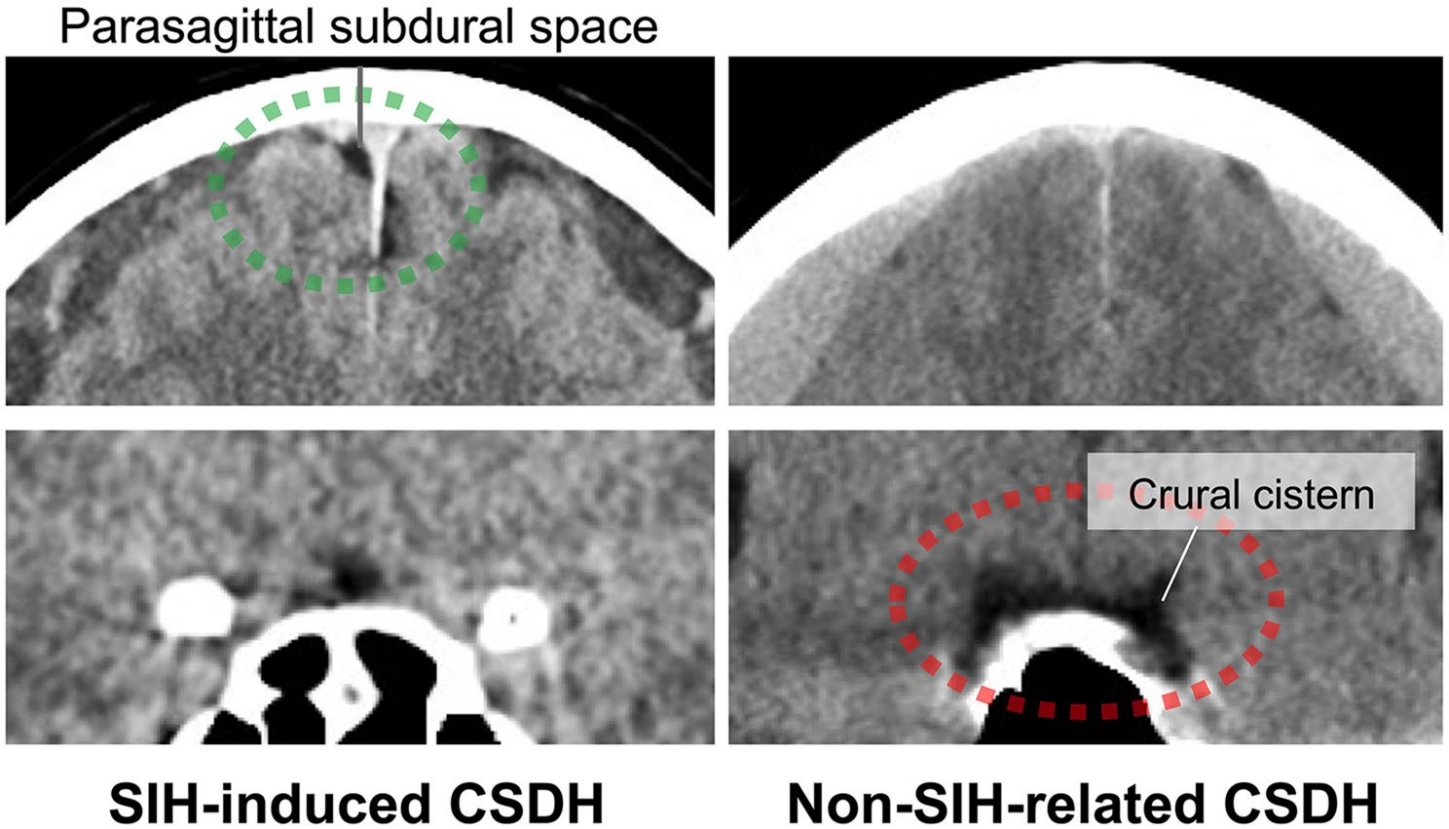
Representative coronal CT image of bilateral CSDH cases of SIH-induced and non-SIH-related

Therefore, early and accurate diagnosis of SIH-induced CSDH is challenging and, at the same time, essential to optimize treatment and avoid serious complications. Although MRI is the gold standard for detecting CSF leaks, head computed tomography (CT) remains the most widely accessible imaging modality in clinical practice. However, its diagnostic utility has been limited by the lack of objective and quantitative markers. To address this gap, we propose the parasagittal subdural space (PSS), defined as the space between the cerebral cortex and the falx cerebri, as a novel quantitative imaging marker for SIH-induced bilateral CSDH. In non-SIH-related CSDH, mass effect from the hematoma typically compresses the medial cerebral hemispheres and obliterates the PSS.In contrast, in SIH-induced cases, cerebrospinal fluid leakage from the spinal canal may cause downward traction of the brain, creating a potential subdural space between the dura mater and the cortical surface [23, 27]. This potential space may preferentially persist along the parasagittal region, resulting in preserved cortical sulci despite the presence of CSDH (Fig. 1). Consistent with previous reports describing downward displacement of the brain and stretching of bridging veins in SIH, this finding may represent a novel and pathophysiologically plausible imaging feature of SIH-induced CSDH [7–9]. By quantifying this space, we aim to provide clinicians with an objective and reproducible tool to improve the diagnostic accuracy of SIH-induced CSDH in routine CT interpretation.

## Materials and methods

### Patient population and data collection

Medical records from January 2008 to May 2023 at our University Hospital were retrospectively reviewed. Because our institution is a tertiary referral center, most patients with uncomplicated CSDH are treated at community hospitals in our area. Therefore, this study mainly consisted of referred cases with atypical or complicated features. The diagnosis of SIH was established when clinical symptoms were suggestive of intracranial hypotension (e.g., orthostatic headache, nausea, or neck stiffness), and whole-spine MRI demonstrated evidence of CSF leakage. Additional imaging to rule out SIH was not routinely performed in patients who had no clinical suspicion of CSF leakage. Specifically, whole-spine MRI was omitted in patients with a clear history of head trauma and without typical orthostatic headache, whereas it was performed in all other patients to exclude SIH.

### Inclusion and exclusion criteria

Patients were included if they (1) were hospitalized with a diagnosis of CSDH, (2) had available preoperative head CT imaging, and (3) presented with bilateral CSDH on initial CT, which is often diagnostically challenging and may indicate underlying SIH. Patients were excluded if they 1) had acute subdural hematoma, defined as a hyperdense, crescent-shaped, and massive supratentorial hematoma with rapidly developing signs of increased intracranial pressure, *2*) had secondary intracranial hypotension (e.g., due to lumbar puncture or trauma-related dural tear), or 3) had follow-up shorter than one month after surgery.

**Fig. 2:**
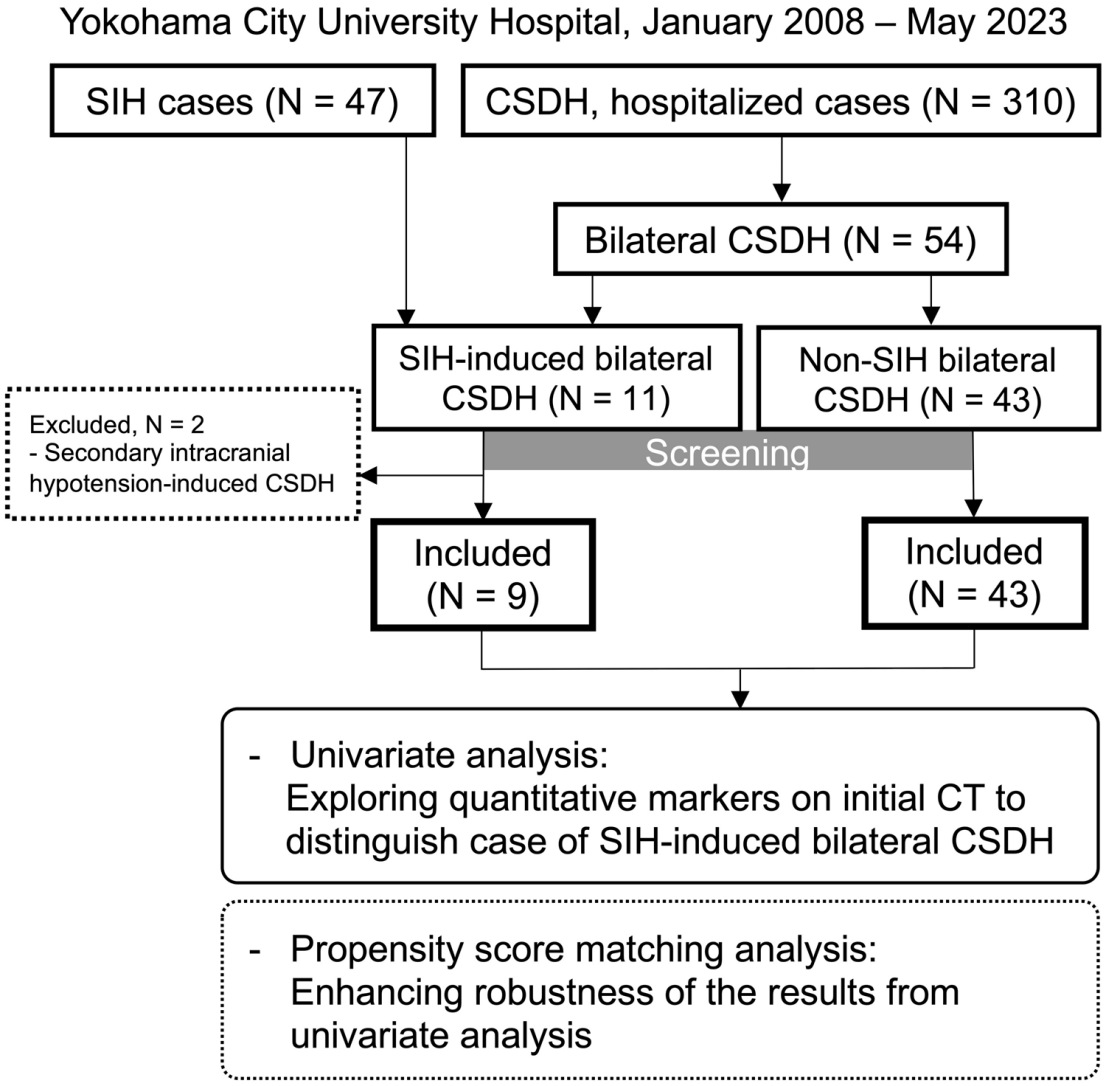
Overview of patient selection and analysis. The diagram illustrates the retrospective review process of 310 hospitalized cases of chronic subdural hematoma (CSDH) at our institution. From these, 54 bilateral CSDH cases were identified and further classified into SIH-induced (*n* = 11) and non-SIH (*n* = 43) groups. Two cases of secondary intracranial hypotension were excluded, leaving 9 SIH-induced cases for inclusion in the study. Both groups underwent univariate analysis to explore quantitative markers on initial CT scans and propensity score matching to enhance the robustness of these results

We analyzed consecutive 310 hospitalized cases of CSDH during this period. Among them, 54 were bilateral and thus eligible for further analysis. Forty-three were classified as non-SIH-related cases, and 11 as SIH-induced CSDH. Among these, two secondary IH cases were excluded, leaving nine patients included as SIH-induced CSDH (Fig. 2).

### Treatment and clinical management

Each case was preoperatively classified as either SIH-induced or non-SIH-induced.

Patients with SIH-induced CSDH were primarily managed conservatively, with a single EBP attempted if symptoms worsened, while non-SIH-related cases were treated according to standard CSDH protocols (e.g., burr-hole drainage). In patients diagnosed with SIH, EBP was performed at the T1/2 and L2/3 levels when thoracic CSF leakage was detected, whereas those without thoracic involvement received EBP solely at the L2/3 level. Age, sex, clinical symptoms, and imaging findings were reviewed, along with clinical course and treatment outcomes. “Recurrence” was defined as clinical and/or radiological deterioration within at least one month of follow-up. Presence of “CSF leakage,” defined as an extradural fluid collection, was assessed on MRI. The cause of CSF leakage was not always identifiable. One patient had vertebral osteophytes unrelated to the leakage site, and no cases involved dural tears or spinal arteriovenous fistulas. Thus, the underlying cause of SIH remained unknown in most patients.

### Ethics and reporting standards

This study utilized predefined imaging definitions and protocols, which were established prior to data collection to ensure consistency and reliability. We adhered to the STARD (Standards for Reporting of Diagnostic Accuracy Studies) guidelines for reporting diagnostic accuracy [28], ensuring full compliance with international reporting standards.

Methods, including the design, execution, and analysis of our retrospective diagnostic study, were conducted in alignment with these standards to ensure transparency, reproducibility, and validity. No indeterminate results were encountered in either the index test (PSS measurement) or the reference standard (MRI diagnosis of CSF leakage). Had such cases occurred, they would have been excluded from quantitative analyses and documented in the study flow diagram (Fig. 2). The study was conducted in accordance with the Declaration of Helsinki and was approved by the Institutional Review Board of Yokohama City University (#2023 − 1235). The requirement for informed consent was waived using an opt-out method, whereby study information was made publicly available, allowing individuals the opportunity to decline participation.

### Image data processing and quantitative analysis

For radiological examination, head CT was performed at admission. Hematoma thickness within 2 mm was defined as symmetric, based on predefined criteria established before the study commenced. For the assessment, qualification of the images and quantification using ImageJ software (Ver.1.54f) were performed in a blinded manner by a single researcher (TT). Radiological and clinical data were statistically analyzed by other researchers (HT and TH).

As qualitative markers, obliteration of the crural-and-ambient cistern and cerebral sulci in the interhemispheric fissure (IHF), and slit-like ventricles in head CT were assessed, as previously described [3, 29]. As quantitative markers, the volume of the hematoma, volume of residual cisterns in the parasagittal sinus, and cerebellar ptosis were also examined. Obliteration of the cerebral sulci in the IHF was evaluated on a coronal CT image showing the foramen of Monro. Cerebellar ptosis was defined as ptosis >5 mm on sagittal section, according to predetermined guidelines [30]. Since CSDH has a variety of imaging characteristics and not always homogeneous [14, 31–33] and recent studies have suggested that hematoma type and maturation grade may be associated with pathogenesis [14, 31, 34], hematomas were classified based on architecture and density: Grade 1 (immature; laminar and homogeneous-high), Grade 2 (intermediate; separated and homogeneous-iso), and Grade 3 (mature; trabecular and homogeneous-low). This classification deviates from the typically universally used Hounsfield Unit measurements in CT imaging, in favor of a more detailed and study-specific approach established prior to data collection [14, 35]. Age was compared in the patients with patent and obliterated sulci in IHF, based on pre-specified criteria for statistical analysis.

### Propensity score matching (PSM)

To enhancing robustness of the results from above mentioned analyses and to account for potential confounders such as age and gender, propensity score matching was employed (Fig. 2). Patients with SIH-induced bilateral CSDH were matched to patients with non-SIH-related bilateral CSDH based on their age and sex using a 1:1 nearest neighbor matching algorithm without replacement. After matching, no significant differences were observed between the two groups in terms of age or sex (*P* > 0.05), confirming adequate covariate balance and reduced heterogeneity between the matched cohorts (Table 3).

### Receiver operating characteristic (ROC) curve analysis

In this diagnostic accuracy analysis, measurement of the PSS on head CT was regarded as the index test, and the diagnosis of SIH confirmed by clinical findings and whole-spine MRI was considered the reference standard. Sensitivity and specificity were calculated to determine the optimal cut-off value for the parasagittal space volume that best discriminates between SIH-induced and non-SIH CSDH. The Area Under the Curve (AUC) was used to evaluate the overall diagnostic accuracy of the parasagittal space volume measurement. The selected cut-off was established through Youden’s Index to maximize the diagnostic potential.

### Statistical analyses

**Fig. 3:**
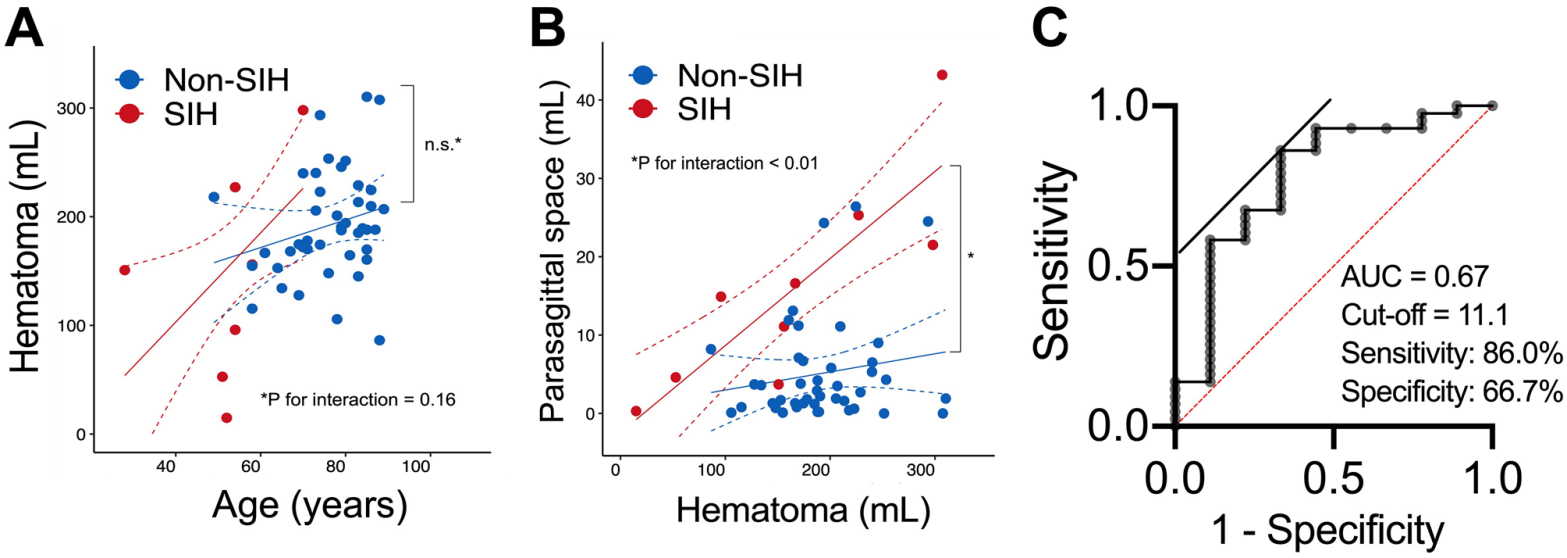
Scatter plot of the age and the volume of hematoma (**A**) and the volume of hematoma and the parasagittal subdural space (**B**) in the groups with SIH (red) and without SIH (blue). The solid and dashed lines show the estimated linear slopes and 95% confident intervals in the unadjusted regression model, respectively. (**C**) ROC analysis identified an exploratory cut-off of 11.1 mm², which yielded a sensitivity of 86% and a specificity of 66.7% (*P* = 0.009)

All statistical analyses were performed using GraphPad Prism (Ver.9.2.0) or R version 4.2.0 (R Foundation for Statistical Computing, Vienna, Austria; URL: https://www.R-project.org). For the univariate analyses, a Mann-Whitney U test was used to compare continuous variables. Fisher’s exact test and the chi-square test were performed for categorical variables in 2 × 2 and 2 × 3 contingency tables, respectively. OR and 95% CI were determined. Linear regression models were used to investigate the association between the age and hematoma volume (Fig. 3A), and the hematoma volume and the parasagittal subdural space (PSS) (Fig. 3B). This involved incorporating a multiplicative interaction term with a group indicator comparing ‘with’ and ‘without’ SIH (i.e., [hematoma volume] * [with vs. without SIH]). Model 1 was unadjusted, whereas Model 2 was adjusted for age and sex. We assessed the model fitness using the F-statistic, which was calculated by comparing the residual sum of squares in the model that included the interaction term to the model without the interaction term, thus evaluating the significance of the interaction term in terms of goodness of fit. We then estimated a separate linear slope for the age versus hematoma volume, and the PSS versus hematoma volume for each group (i.e., with or without SIH).

For ROC curve analysis, the cut-off value for the parasagittal space that optimally distinguished between the two conditions was determined, with sensitivity and specificity values calculated at this threshold. The AUC provided a measure of the test’s ability to correctly classify those with and without SIH-induced CSDH. Accordingly, the ROC analysis quantified the diagnostic performance of the CT-based PSS measurement (index test) against the MRI-confirmed diagnosis of SIH (reference standard).

Data are expressed as mean ± standard deviation. Statistical significance was set at *P* < 0.05. Data supporting the findings of this study are available from the corresponding author upon reasonable request.

## Results

Of hospitalized 54 patients with bilateral CSDH, 43 were non-SIH-related, and the remaining 11 were SIH-induced. Among them, 2 cases were excluded because the IH were secondary, and finally 9 cases were included as SIH-induced CSDH (Fig. 2). In total 52 bilateral CSDH, the average age was 72.5 years and thirty-seven cases are male (71.2%). On radiological assessment, the mean volume of hematoma and PSS were 187.2 mL and 6.9 mL, respectively. Obliteration of the crural-and-ambient cistern and cerebral sulci in the interhemispheric fissure (IHF), cerebellar ptosis, and slit-like ventricles were observed in 21.2%, 59.6%, 3.8%, and 86.5%, respectively (Tables 1 and 2, S1).

**Table 1:** Characteristics of the included patients. Univariate analyses for the difference between the groups bilateral chronic subdural hematoma (CSDH) with and without spontaneous intracranial hypotension syndrome (SIH). Groups of “with SIH” and “without SIH” were compared via Mann-Whitney U test, fisher’s exact- or Chi-squared test as appropriate. CI, confidence interval. OR, odds ratio. BMI, body mass index. IHF, interhemispheric fissure

Variables	Overall	Bilateral CSDH			
		SIH	Non-SIH	Univariate analysis	
				OR [95%CI]	p-value
Number of patients (%)	52	9 (17.3)	43 (82.7)		
Age (year, mean ± SD)	72.5 ± 12.6	54.7 ± 11.0	76.2 ± 9.2		< 0.001
Male cases (%)	37 (71.2)	5 (55.6)	32 (74.4)	0.43 [0.097, 1.63]	0.42
BMI (kg/m^2^, mean ± SD)	22.8 ± 3.5	22.3 ± 4.7	21.3 ± 3.9		0.64
Hypertension (%)	16 (30.8)	1 (11.1)	15 (34.9)	0.23 [0.020, 1.53]	0.24
Diabetes (%)	9 (17.3)	2 (22.2)	7 (16.3)	1.47 [0.26, 8.14]	0.65
Smorking (%)	5 (9.6)	0 (0)	5 (11.6)	0 [0, 3.65]	0.57
Orthostatic headache (%)	8 (40.0)	6 (66.7)	1 (2.3)	84.0 [6.86, 966.0]	< 0.001
Trauma history (%)	11 (21.2)	2 (22.2)	9 (20.9)	1.08 [0.20, 5.40]	>0.99
Decreased level of consciousness (%)	10 (19.2)	4 (44.4)	6 (14.0)	4.93 [1.19, 21.2]	0.057
Hematoma maturation grade (%)					0.69
1: Immature_Homogenous-high/Laminar	7 (13.5)	2 (22.2)	5 (11.6)		
2: Intermediate_Homogenous-iso/Separated	25 (48.1)	3 (33.3)	22 (51.2)		
3: Mature_Homogenous-low/Trabecullar	15 (28.8)	4 (44.4)	11 (25.6)		
1+2	2 (3.8)	0 (0)	2 (4.7)		
1+3	1 (1.9)	0 (0)	1 (2.3)		
2+3	2 (3.8)	0 (0)	2 (4.7)		
Initial volume of hematoma (mL; mean ± SD)	187.2 ± 60.5	163.2 ± 95.4	192.2 ± 48.7		0.25
Volume of parasagittal subdural space (mL; mean ± SD)	6.9 ± 2.3	15.0 ± 12.5	5.1 ± 6.5		0.007
Obliteration of ambient cistern (%)	11 (21.2)	7 (77.8)	4 (9.3)	34.1 [5.40, 179.5]	< 0.001
Obliteration of cerebral sulci in IHF (%)*	31 (59.6)	3 (33.3)	28 (65.1)	0.27 [0.068, 1.08]	0.13
Cerebellar ptosis (%)	2 (3.8)	2 (22.2)	0 (0)	Infinity [2.37, Infinity]	0.027
Slit-like ventricles (%)	45 (86.5)	9 (100)	36 (83.7)	Infinity [0.47, Infinity]	0.33

OR: odds ratio, SD: standard deviation, BMI: body mass index, IHF: interhemispheric fissure, N/A: not applicable

*In cases where one side of the IHF is preserved and the other is obliterated, findings from the side with the larger hematoma was adopted

**Table 2:** Details of the bilateral CSDH cases with SIH. BMI: body mass index, HT: hypertension, DM: diabetes, IHF: interhemispheric fissure, C: cervical, T: thoracic, EBP: epidural blood patch, ID: irrigation and drainage, co: Conservative treatment

								Image findings											
Case No.	Age, Sex	BMI	HT	DM	Smorking	Headache	Orthostatic headache	Progressive deterioration of consciousness	Hematoma type	Hematoma maturation grade	Volume of hematoma (mL)	Volume of parasagittal subdural space (mL)	Obliteration of crural-and-ambient cistern	Obliteration of cerebral sulci in IHF	Cerebellar ptosis	Slit-like ventricles	Level of CSF leakage	Treatment	Outcome at discharge
1	54, M	25.2	-	-	-	+	+	-	Trabecullar	3	95.9	14.9	+	-	-	+	C 1/2	EBP, ID	Good
2	58, M	22.1	-	-	-	+	+	+	Hemogenous-iso	2	156.0	11.1	+	+	-	+	C 3/4	EBP, ID	Good
3	51, F	18.7	-	-	-	+	+	-	Hemogenous-low	3	52.7	4.6	+	-	-	+	T 4/5	Co	Good
4	52, F	30.4	+	+	-	+	+	+	Laminar	1	14.9	0.3	+	+	-	+	C 3/4	EBP, ID	Good
5	61, F	17.6	-	-	-	+	+	-	Trabecullar	3	166.7	16.6	+	-	-	+	C 5/6	EBP	Good
6	64, M	20.6	-	+	-	+	-	-	Laminar	2	306.9	43.2	-	-	-	+	C 2/3	ID, EBP	Good
7	28, M	29.4	-	-	-	+	+	-	Hemogenous-iso	2	150.8	3.7	+	+	+	+	C 1/2	EBP, ID	Good
8	54, M	21.5	-	-	-	+	-	-	Hemogenous-low	3	227.1	25.3	+	-	+	+	T 6-8	EBP, ID	Good
9	70, F	15.6	-	-	-	+	+	-	Hemogenous-iso	2	298.0	21.5	-	-	-	+	C 2/3	EBP, ID	Good

BMI: body mass index, HT: hypertension, DM: diabetes, IHF: interhemispheric fissure, C: cervical, T: thoracic, EBP: epidural blood patch, ID: irrigation and drainage, Co: conservative treatment

CSF leakage was confirmed via magnetic resonance imaging (MRI) in all nine patients with SIH-induced bilateral CSDH, and found in the spinal region, but not in the skull-base (Table 2). Seven patients were initially treated with conservative therapy, followed by an EBP. Among them, two patients presented with progressive deterioration of consciousness, thus, the observation periods of conservative therapy were shorter. One patient initially underwent surgical drainage because SIH was not suspected at presentation. Subsequently, the bilateral CSDH deteriorated, and EBP was performed. Another case was treated conservatively. Of the seven patients initially treated with EBP, six required surgical drainage. However, one patient improved without drainage. All the patients with SIH-induced bilateral CSDH demonstrated good outcomes at discharge.

In the bilateral CSDH with SIH group, mean age was significantly lower compared with that of the without SIH group (Table 1). The rates of presenting orthostatic headache were significantly higher in the SIH group than non-SIH.

In radiological analyses, the initial volume of hematoma was not significantly different between the groups (Table 1). Additionally, in the linear regression models, association was not significant between the hematoma volume and the interaction effect between age and the group indicator in the unadjusted and adjusted models (Fig. 3A). This means that the age-adjusted hematoma volume was not significantly different between the two groups. In turn, the PSS volume was significantly greater in the SIH group than that in non-SIH. When both groups were compared in linear regression models, a significant association between the PSS and the interaction effect between hematoma volume and the group indicator was observed in the unadjusted and adjusted models (Fig. 3B). When the F-statistic for models was compared, the interaction term between hematoma volume and the group indicator significantly contributed to model fit. These results demonstrate that the presence of SIH strengthened the association between the volume of the PSS and volume of the hematoma. In other words, the slopes were significantly different between the groups (Fig. 3B). Standardized linear slopes (95% CIs) for PSS versus hematoma volume for the with and without SIH groups were 0.76 (0.44–1.08) and 0.16 (-0.13-0.45), respectively. After adjusting for covariates including age and sex, the estimated slopes were 0.65 (0.33–0.98) and 0.06 (-0.23-0.35), respectively. Obliteration of the crural-and-ambient cistern and cerebellar ptosis were also significantly higher in the SIH group.

We performed a PSM based on age and gender, analyzing 9 pairs of patients with SIH-induced and non-SIH bilateral CSDH (Table 3). After propensity score matching, baseline characteristics including age and sex were well balanced between the SIH and non-SIH groups (*P* > 0.05), indicating successful matching. Notably, a consciousness decreasing was significantly more prevalent in the SIH-induced group. Radiological assessments showed no significant differences in the initial volume of hematoma between the groups. However, the PSS volume was significantly larger in the SIH-induced group, similar to the result of linear regression model (Fig. 3B). Furthermore, obliteration of cerebral sulci in the IHF was also significantly common in the SIH-induced group.

ROC curve analysis was utilized to determine the diagnostic utility of PSS volume in differentiating between SIH-induced and non-SIH CSDH (Fig. 3C). The optimal cut-off value for PSS volume was established at 11.1 mL, with a sensitivity of 86.0% and a specificity of 66.7% (*P* = 0.009). The Area Under the Curve was calculated to be 0.67, indicating a moderate diagnostic accuracy of PSS volume measurements in this context.

**Table 3:** Propensity score matching analysis for the difference between the groups bilateral CSDH with and without SIH. Groups of “with SIH” and “without SIH” were compared via Mann-Whitney U test, fisher’s exact- or Chi-squared test as appropriate. BMI, body mass index. IHF, interhemispheric fissure. PSM, propensity score matching analysis

Variables	PSM
	*p*-value
Age	0.97
Sex	>0.99
BMI	0.75
Hypertension	0.35
Diabetes	0.17
Smorking	N/A
Orthostatic headache	0.18
Trauma history	0.33
Decreased level of consciousness	0.035
Initial volume of hematoma	0.89
Volume of parasagittal subdural space	0.010
Obliteration of ambient cistern	0.17
Obliteration of cerebral sulci in IHF	0.004
Cerebellar ptosis	0.17
Slit-like ventricles	0.47

OR: odds ratio, SD: standard deviation, BMI: body mass index, IHF: interhemispheric fissure, N/A: not applicable, PSM; propensity score matching analysis

### Illustrative cases

#### Case 8: bilateral CSDH with SIH

**Fig. 4:**
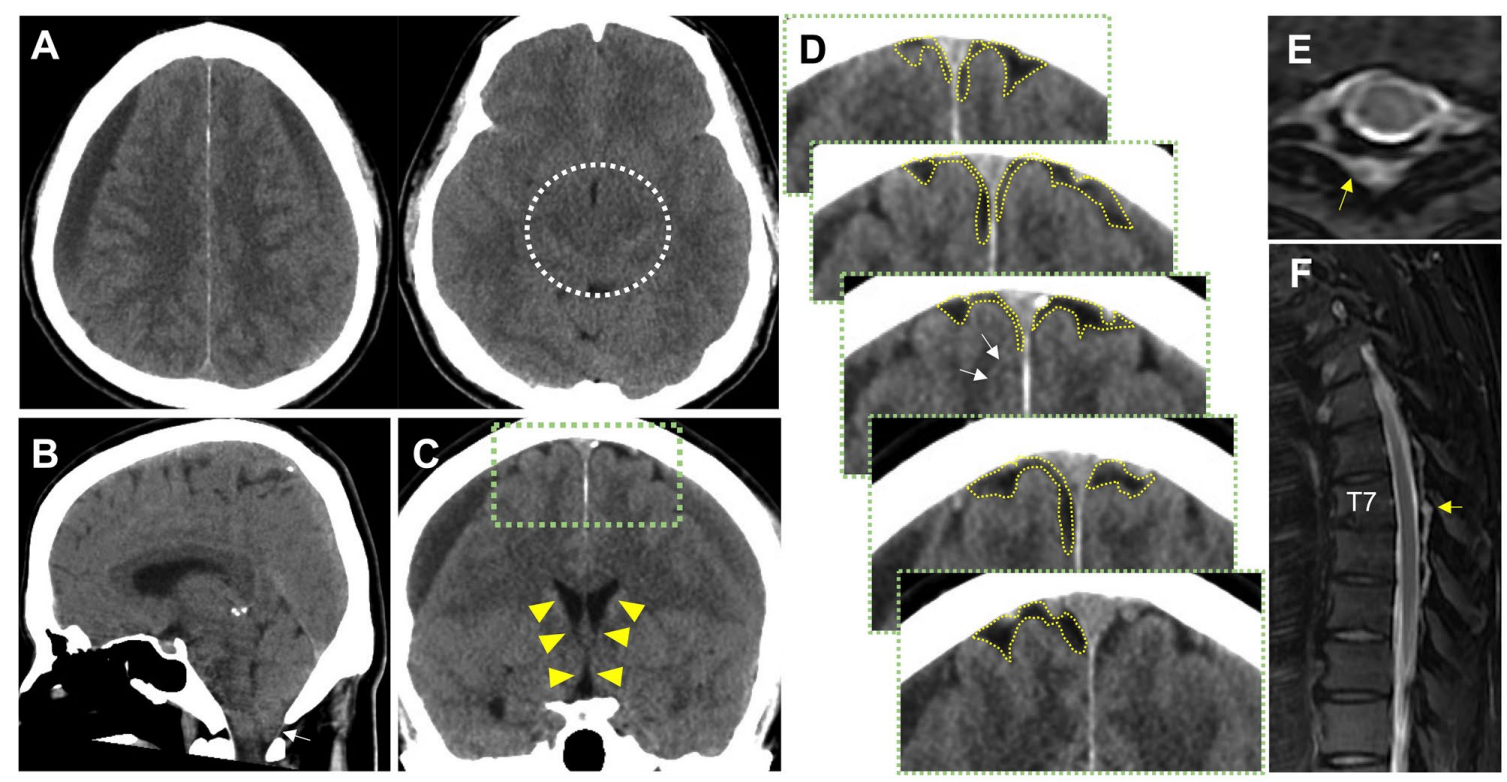
Initial CT findings of a case of bilateral CSDH with SIH (Case 8: male patient in his 50s, with headache but not orthostatic). Head CT showing (**A: axial**) (Left) symmetric bilateral CSDH, (Right) obliteration of crural-and-ambient cisterns (white dotted circle), (**B: sagittal**) cerebellar ptosis (white arrow), (**C: coronal**) slit-like ventricles (yellow arrowheads), (**D**) patency of cerebral sulci in the IHF (white arrows), and PSS (yellow dotted area) indicating possible SIH-induced bilateral CSDH. (**E**,** F**) T2-weighted image of spinal MRI showing high intensity in the epidural space at the level of T6-9, “CSF leakage (yellow arrow)”, proving SIH-induced CSDH

**Fig. 5:**
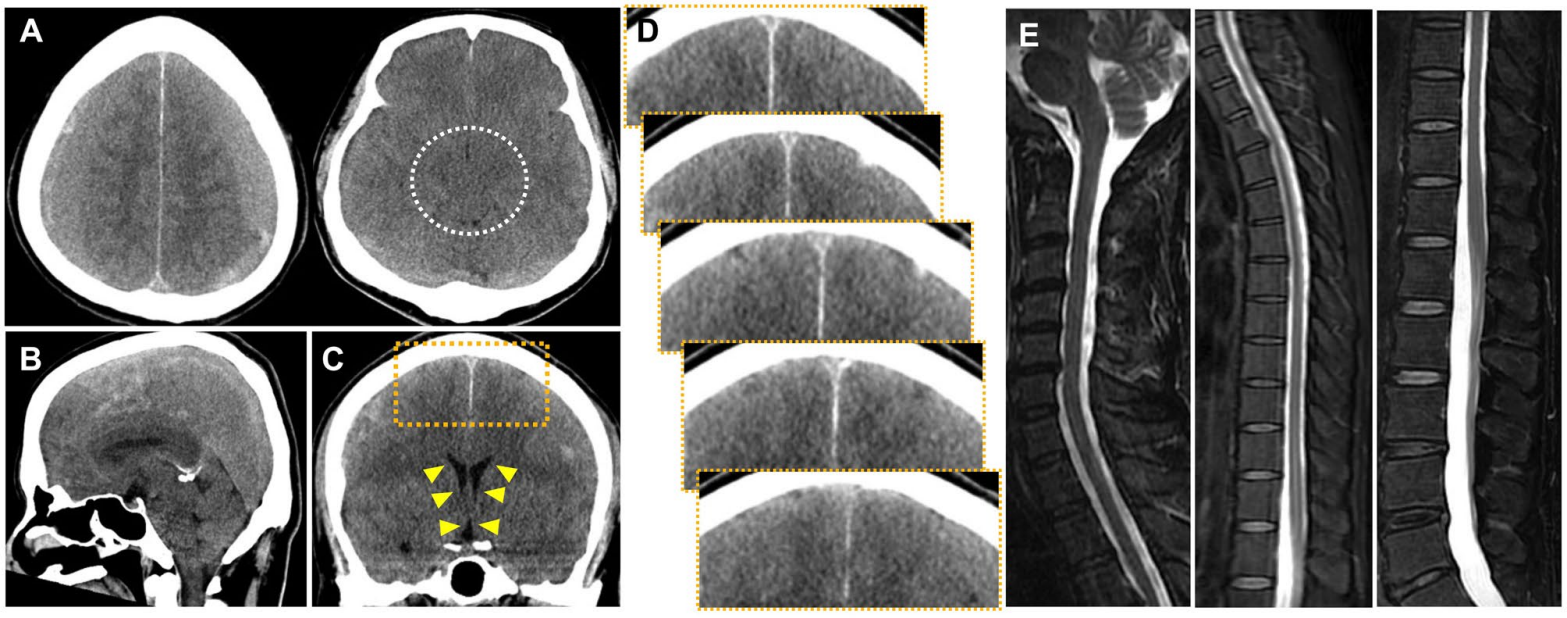
Initial CT findings of a case of bilateral CSDH without SIH (Case 20: male patient in his 40s, with two-month history of gradually worsening orthostatic headache). Head CT showing (**A: axial**) (Left) symmetric bilateral laminar and iso-hyperdense CSDH, which was categorized in Grade 1, (Right) obliteration of crural-and-ambient cisterns (white dotted circle), and (**C: coronal**) slit-like ventricles (yellow arrowheads). (**B: sagittal**) However, unlike SIH-induced case, cerebellar ptosis was not shown. Moreover, (**D**) cerebral sulci in the IHF and PSS were obliterated indicating possible non-SIH-related bilateral CSDH. (**E**) MRI indicated that there was lacking CSF leakage, diagnosing non-SIH-related CSDH

A man in his 50s without any history of trauma presented with headache, which was not orthostatic, that gradually worsened. Bilateral CSDH was found on head CT, and the patient was admitted (Fig. 4A; Table 2). His CT showed obliteration of the crural-and-ambient cisterns (Fig. 4B), cerebellar ptosis (Fig. 5C), and slit-like ventricles (Fig. 5D). In contrast, the cerebral sulci in the IHF were patent on the coronal head CT (Fig. 5E; white arrows), indicating possible SIH. T2-weighted spinal MRI revealed high intensity in the epidural space at the level–T6-9 (Fig. 5F, G), indicating SIH-induced CSDH. As conservative therapy failed, EBP was performed at the T1/2 level and L2/3 level, and as a result, the patient’s headache improved. However, one week later, the patient presented with headache, and CT showed hematoma growth. Nevertheless, the cerebral sulci in the IHF were obliterated and the PSS was tightened. All clinical symptoms improved after the surgical drainage (Figure S1).

### Case 20: bilateral CSDH without SIH

A man in his 40s with no history of trauma presented with orthostatic headache that gradually worsening in two months. Head CT revealed symmetric bilateral CSDH (Fig. 5A), obliteration of the crural-and-ambient cisterns (Fig. 5B), and slit-like ventricles (Fig. 5D), suggesting SIH-induced bilateral CSDH; although, he lacked cerebellar ptosis (Fig. 5C). However, the cerebral sulci in the IHF and the PSS were completely obliterated (Fig. 5E). Moreover, MRI indicated a lack of CSF leakage (Fig. 5F-H), indicating non-SIH-related bilateral CSDH. The headache improved after surgical drainage, and the patient was discharged without complications.

## Discussion

This case-control study assessed SIH-induced versus non-SIH bilateral CSDH, highlighting initial CT [9]. Our findings confirm that SIH-induced bilateral CSDH occurs predominantly in younger individuals and often presents with orthostatic headaches. It is also characterized by a larger PSS and distinct radiological features. These quantitative indicators, critical for diagnosis, include obliteration of crural-and-ambient cisterns, cerebral sulci in IHF, and cerebellar ptosis, setting them apart from the more qualitative assessments commonly used (Fig. 3B; Tables 1 and 3). Although other CT findings such as sulcal effacement in the interhemispheric fissure or cerebellar ptosis have also been reported as supportive signs of SIH, we focused on the PSS because it provides a quantitative, objective, and reproducible measure that can be readily assessed on routine axial or coronal CT scans. Unlike sulcal or posterior fossa changes, which often rely on subjective interpretation and can be subtle, PSS offers a more standardized metric that may be especially useful for non-specialists and in emergency settings.

Despite its common association with aging and minor trauma, CSDH may not be as benign as previously thought, particularly in elderly patients [36, 37]. On the other hand, SIH, which was once believed to be rare, can present with a variety of symptoms, making diagnosis challenging [2, 6, 37–44]. Key symptoms like orthostatic headache somewhat aid in diagnosis [5, 45], but timely and accurate identification is crucial. This is particularly important because there are often delays with MRI, the standard diagnostic tool [14, 41, 46]. Therefore, having reliable quantitative marker on head CT could lead to faster recognition of the condition [47, 48]. In fact, some reports have indicated harmful outcomes from surgical interventions used to treat misdiagnosed cases of SIH-induced CSDH [22, 23, 45].

Among our SIH-related CSDH patients, one case was managed successfully with EBP alone, without requiring surgical drainage. In contrast, five patients ultimately underwent burr-hole drainage. This may be explained by the increase in intracranial pressure after EBP in patients with initially low-pressure SIH, which made the hematoma symptomatic even though its size remained unchanged (Case 8). In the present study, almost all patients were diagnosed with SIH and treated with an EBP; however, surgical evacuation of CSDH was still required in some cases. Nevertheless, it is crucial to establish an accurate diagnosis of SIH-induced CSDH. If burr-hole surgery is performed without recognizing the underlying SIH and without prior EBP, intracranial hypotension may be further exacerbated, leading to neurological deterioration or even fatal outcomes. Therefore, careful identification and appropriate management of SIH are essential before considering surgical intervention for CSDH.

SIH, including cases that lead to CSDH, is generally treated conservatively, similar to non-emergent CSDH cases [6, 49]. Treatment often includes an EBP to ameliorate IH [5]. In Case 8, the application of EBP resulted in change in PSS and crural-and-ambient cisterns on follow-up head CT, which suggested a resolution of SIH (Figure S1). In our study, 17.3% of bilateral CSDH cases were linked to SIH, with no significant age-related differences in hematoma volume or type, aligning with literatures [2, 5, 9, 20, 23]. In contrast, CSDH usually develops in the elderly; [37] therefore, in this study, the age of patients with non-SIH CSDH was significantly higher than that with SIH-induced CSDH (Table 2). Our use of both linear regressions and PSM helped to confirm these differences, highlighting the unique pathophysiological profile of SIH (Figs. 3A-B and 4).

History of preceding trauma can be a potential risk factor for both SIH and CSDH. Nevertheless, Takahashi et al. reported 55 cases of CSDH with SIH, noting trauma history in just 6 cases (10.9%). This suggests that a history of preceding trauma is not a common factor in CSDH case with SIH (Table 4) [23, 50, 51]. Present study also showed no significant difference between the groups. As causes of bilateral CSDH, renal dysfunction or use of antithrombotic drug are suggested, but there were not such cases in current study [26, 52].

**Table 4:** Reported diagnostic features of SIH-induced CSDH. This table summarizes previously reported clinical and radiological features that help differentiate SIH-induced CSDH from non-SIH-related cases. The features are categorized by patient characteristics, clinical history, and radiological markers. For each feature, the imaging modality, diagnostic definition, qualitative or quantitative classification, reported sensitivity and specificity (when available), and supporting literature are provided. Quantitative parameters, such as fluid collection depth and parasagittal subdural space area, are included to highlight their diagnostic potential. The current study contributes new findings regarding the parasagittal subdural space as a quantitative marker

Categories	Diagnostic feature	Modality	Definition	Qualitative or Quantitative	Sensitivity, %	Specificity, %	Note	Author, Year
Patients characteristics	Younger age			Qualitative			Four cases of unilateral CSDH with coma, with av. 53 y.o.	Osada et al. 2020
	Younger age		≤55 years old	Qualitative	100	88.9		Kim JH et al. 2019
Clinical history	No significant trauma history			Qualitative				Nakajima et al. 1996, Murakami et al. 2000, Takahashi et al. 2007, Souirti et al. 2009, Umebayashi et al. 2011, De Carvalho et al. 2013, Kim JH et al. 2019, Osada et al. 2020, Lee et al. 2024
Radiological markers	bilateral CSDH	CT/MRI		Qualitative			Commonly present in a certain percentage of ordinary CSDH cases	Sipe et al., 1981, Nakajima et al., 1996, Murakami et al. 2000, Souirti et al., 2009
	Transtentorial herniation	CT/MRI		Qualitative				Chi et al. 2007, Osada et al. 2020, Pfeiffer 2022
	Brain sagging	CT	Obliteration of cisternal spaces around brainstem: cerebellomedullary, quadrigeminal, prepontine, and basal cisterns	Qualitative				Kim JH et al. 2019
	Small amount of fluid collection	CT	≤22 mm of total depth	Quantitative	100	81.5		Kim JH et al. 2019
					100	55.8		Current study
	Pseudo-subarachnoid hemorrhage	CT	Obliteration of basal cistern with high-density lesions	Qualitative				Kim JH et al. 2019
	Parasagittal subdural space	CT	≤11.1 mm2 of the space	Quantitative	86	66.7		Current study

While orthostatic headaches are a hallmark of SIH, their presence alone is not definitive for diagnosis. Our study found this symptom to be significantly more prevalent in the SIH group, yet not universally present, underscoring the insufficiency of headache as a sole diagnostic marker. A recent systematic review reported that headache and orthostatic headache were the most frequent symptoms of SIH (98.6% and 96.3%) [5]. However, since some patients with SIH do not experience headache, the authors indicated that a diagnosis of SIH should not be excluded based on the absence of orthostatic headache. Other supportive markers need to be identified for accurate diagnosis, and for this purpose, CT may be preferable due to better accessibility than MRI.

Normally, the volume of the subdural space increases with age [45, 46]. Therefore, the relatively large PSS observed in younger SIH patients may reflect a distinct pathophysiological mechanism. In this study, PSS was found to be significantly larger in SIH-induced bilateral CSDH compared to non-SIH cases. This difference remained evident even after adjusting for age using PSM (Table 3). PSS showed a positive correlation with hematoma volume in the SIH group, unlike in non-SIH cases (Fig. 3B), supporting its utility as a reliable quantitative marker for SIH-related cases. Furthermore, ROC analysis of PSS yielded a sensitivity of 86.0% and a specificity of 66.7% (Fig. 3C), indicating good diagnostic performance. Based on these results, we propose that PSS should be routinely measured in initial CT scans of bilateral CSDH cases. Especially when orthostatic headache is present, the preservation of PSS may strongly indicate SIH as the underlying cause. Although the ROC analysis of PSS demonstrated good diagnostic performance, combining PSS with additional imaging parameters, such as obliteration of the crural-and-ambient cisterns or cerebellar ptosis, could further enhance diagnostic accuracy. Future studies with larger cohorts may enable the development of a multi-parameter diagnostic model that integrates both quantitative and qualitative CT features to optimize the detection of SIH-induced CSDH. This quantitative marker complements qualitative findings, such as obliteration of the crural-and-ambient cisterns, which may also be helpful in the initial diagnosis of SIH-induced CSDH (Table 2). In bilateral CSDH cases, particularly with orthostatic headache, preserved PSS on initial CT should prompt consideration of SIH as the underlying cause.

A previous study comparing eight SIH-induced and 54 non-SIH CSDH cases reported that SIH-induced CSDH was associated with younger age, absence of comorbidities, and qualitative radiological signs such as pseudo-subarachnoid hemorrhage and brain sagging, defined by obliteration of cisternal spaces around the brainstem [9]. Our findings support these observations and additionally provide quantitative evidence for the “brain sagging” theory by evaluating PSS on CT. While the previous study suggested that smaller hematoma volume is a characteristic feature of SIH-induced CSDH, our analysis found no significant difference in volume between groups (Table 1). However, when measuring hematoma depth at the thickest point with a cut-off of 22.08 mm, we achieved a sensitivity of 100% and specificity of 55.8% (*P* < 0.001). This suggests that hematoma depth may be a useful marker, though less specific than the PSS, which in our analysis showed a sensitivity of 86.0% and specificity of 66.7%. In clinical practice, incorporating such quantitative markers, including PSS and hematoma depth, may improve diagnostic accuracy and aid in better stratification and management of patients. A summary of diagnostic features distinguishing SIH-induced and non-SIH bilateral CSDH is provided in Table 4.

**Fig. 6:**
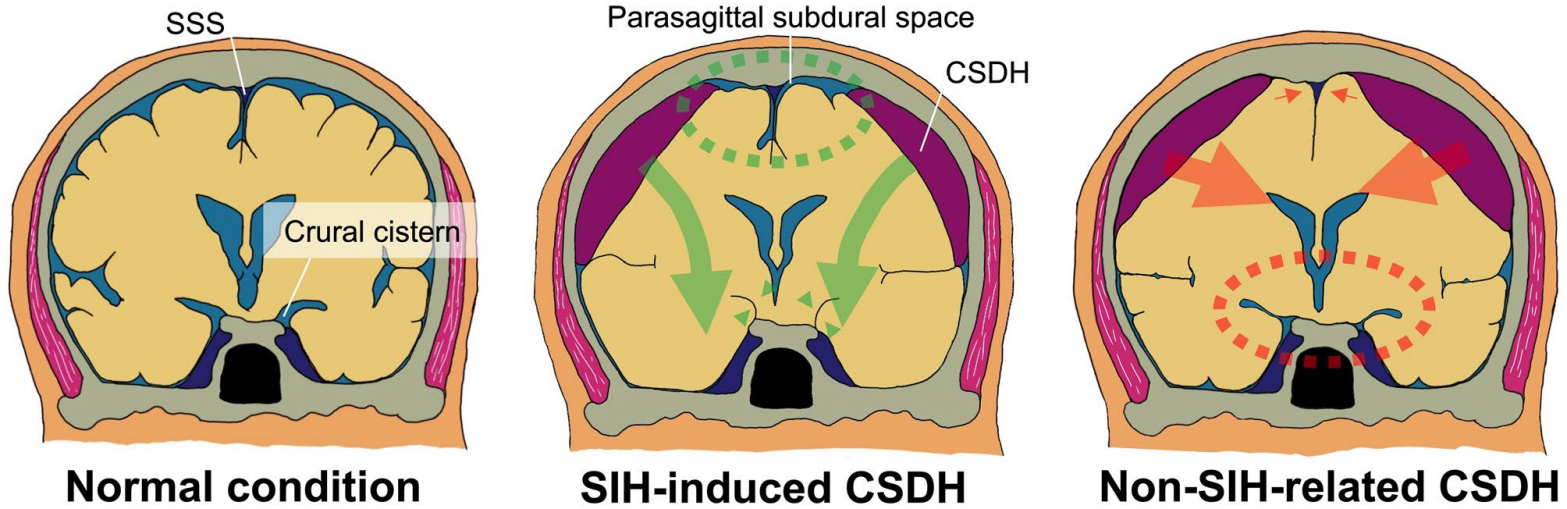
Possible CT-features of SIH-induced and non-SIH-related bilateral CSDH. (Left) Normal condition. PSS (beside SSS) and crural cistern are patent. (Middle) Since bilateral CSDH occurs due to CSF leakage at skull-base or spinal level (green thick-arrows) in SIH-induced case, crural cistern is obliterated (green arrowheads), and PSS is patent (green dotted area). (Right) On the contrary, in non-SIH-related bilateral CSDH, crural cistern is patent (orange dotted area), and PSS is obliterated (orange small-arrows) as hematoma compresses the supratentorial components in midline (orange thick-arrows). SIH: spontaneous intracranial hypotension syndrome, CSDH: chronic subdural hematoma, SSS: superior sagittal sinus

Since these radiological differences were not associated with age or sex (covariate-adjusted regression model, Fig. 3A-B, S1), these features may result from a caudal shift of the intracranial component due to CSF leakage from the skull-base or spinal epidural space (Figs. 1, 4, 5 and 6). According to this theory, the cerebral sulci in the IHF can be obliterated in SIH cases. In fact, this was observed in current PSM analysis.

In recent years, several MRI-based diagnostic scoring systems have been proposed for SIH, incorporating features such as pachymeningeal enhancement, venous engorgement, subdural fluid collection, and brain sagging. While these approaches are undoubtedly valuable, MRI is often less accessible in urgent care settings or in resource-limited environments, leading to delays in diagnosis and treatment. In contrast, head CT is widely available, fast, and cost-effective, making it the first-line modality for many patients presenting with headache or suspected CSDH. Our study suggests that PSS measurement on CT could serve as a practical alternative or adjunct to MRI-based assessments, particularly when timely MRI is not feasible. This underscores the clinical utility of simple, CT-based markers like PSS in real-world settings.

Although our study identified PSS as a single quantitative CT marker with diagnostic value, combining it with additional clinical and radiographic parameters could further improve diagnostic accuracy and clinical applicability. For instance, integrating orthostatic headache, cerebellar ptosis, and cisternal obliteration into a composite diagnostic score may provide a more comprehensive and objective tool for differentiating SIH-induced CSDH from non-SIH cases in daily clinical practice. Future multicenter studies with larger cohorts will be required to validate such an integrated scoring approach and to assess its predictive performance compared with PSS alone.

This study has several limitations. First, as a retrospective single-center study, selection bias cannot be entirely excluded. Second, the number of SIH-induced cases was small (*n* = 9), although this represents one of the largest case series of bilateral SIH-induced CSDH reported to date. Given the rarity of this condition, further validation in larger, ideally prospective, multicenter cohorts is warranted. Third, there was a significant age difference between the SIH and non-SIH groups, which could potentially confound the observed association, despite statistical adjustments. The younger age in the SIH group is consistent with previous reports, but future age-matched studies will be necessary to confirm these findings [9, 13, 23, 53, 54]. Fourth, the proposed PSS cut-off demonstrated high sensitivity (88.9%) but only moderate specificity (66.7%). Whereas this may limit its standalone diagnostic power, we believe that PSS measurement can serve as a useful adjunct marker in the clinical context, particularly when MRI is not readily available. Fifth, this study included only bilateral CSDH cases, which are more commonly associated with SIH, and thus the findings may not be applicable to the extremely rare unilateral SIH-induced CSDH [20, 55]. Sixth, the average hematoma volume in this study was relatively large, particularly in the non-SIH group, potentially affecting mass effect and the detectability of PSS. Therefore, caution should be taken when generalizing these findings to cases with small-volume hematomas [56, 57]. Lastly, although a one-month clinical follow-up was performed, subtle or recurrent SIH-related symptoms such as orthostatic headache may have been underdetected. Additionally, in the non-SIH-related group, undiagnosed CSF leaks, such as from venous fistulas or nerve root sleeve tears, cannot be completely ruled out based on non-enhanced MRI alone [58–60]. Moreover, this study did not specifically evaluate differences in clinical deterioration or outcomes between SIH and non-SIH patients, as it was primarily designed to assess the diagnostic performance of the PSS as an imaging marker. Future studies focusing on longitudinal clinical outcomes may provide further insights into the prognostic relevance of this imaging phenotype.

## Conclusions

This study proposes that a preserved PSS on CT may serve as a novel and practical quantitative marker for diagnosing SIH-induced bilateral CSDH. Despite the limitations in sample size, age balance, and specificity, the findings highlight the potential utility of PSS measurement, particularly in settings where MRI is unavailable. This exploratory study underscores the need for larger, prospective, multicenter investigations to validate the diagnostic performance of PSS and to better integrate it into clinical decision-making algorithms.

## Supplementary Information

Below is the link to the electronic supplementary material.

Supplementary Material 1

Supplementary Material 2

Supplementary Material 3

## Data Availability

The datasets generated and/or analyzed during the current study are available from the corresponding author on reasonable request.

## Abbreviations

SIH: Spontaneous intracranial hypotension syndrome
IH: Intracranial hypotension
CSF: Cerebrospinal fluid
CSDH: Chronic subdural hematoma
CT: Computed tomography
EBP: Epidural blood patch
IHF: Interhemispheric fissure
PSS: Parasagittal subdural space
MRI: Magnetic resonance imaging
PSM: Propensity score matching
ROC: Receiver operating characteristic

## References

[1] Headache Classification Committee of the International Headache Society (IHS) The International Classification of Headache Disorders, 3rd edition. Cephalalgia. 2018, 38(1):1-211. 10.1177/0333102417738202 29368949

[2] Schievink WI, Maya MM, Moser F, Tourje J, Torbati S. Frequency of spontaneous intracranial hypotension in the emergency department. J Headache Pain. 2007;8(6):325–8. 18071632 10.1007/s10194-007-0421-8 PMC3476164

[3] Schievink WI. Stroke and death due to spontaneous intracranial hypotension. Neurocrit Care. 2013;18(2):248–51. 23196352 10.1007/s12028-012-9800-3

[4] Amrhein TJ, Williams JW Jr., Gray L, Malinzak MD, Cantrell S, Deline CR, Carr CM, Kim DK, Goldstein KM, Kranz PG. Efficacy of epidural blood patching or surgery in spontaneous intracranial hypotension: A systematic review and evidence map. AJNR Am J Neuroradiol. 2023;44(6):730–9. 37202114 10.3174/ajnr.A7880 PMC10249694

[5] D’Antona L, Jaime Merchan MA, Vassiliou A, Watkins LD, Davagnanam I, Toma AK, Matharu MS. Clinical Presentation, investigation Findings, and treatment outcomes of spontaneous intracranial hypotension syndrome: A systematic review and Meta-analysis. JAMA Neurol. 2021;78(3):329–37. 33393980 10.1001/jamaneurol.2020.4799 PMC7783594

[6] Ferrante E, Trimboli M, Rubino F. Spontaneous intracranial hypotension: review and expert opinion. Acta Neurol Belg. 2020;120(1):9–18. 31215003 10.1007/s13760-019-01166-8

[7] Schievink WI. Spontaneous spinal cerebrospinal fluid leaks and intracranial hypotension. JAMA. 2006;295(19):2286–96. 16705110 10.1001/jama.295.19.2286

[8] Wang DJ, Pandey SK, Lee DH, Sharma M. The interpeduncular angle: A practical and objective marker for the detection and diagnosis of intracranial hypotension on brain MRI. AJNR Am J Neuroradiol. 2019;40(8):1299–303. 31296521 10.3174/ajnr.A6120 PMC7048482

[9] Kim JH, Roh H, Yoon WK, Kwon TH, Chong K, Hwang SY, Kim JH. Clinical features of patients with spontaneous intracranial hypotension complicated with bilateral subdural fluid collections. Headache. 2019;59(5):775–86. 30985923 10.1111/head.13525

[10] Karibe H, Kameyama M, Kawase M, Hirano T, Kawaguchi T, Tominaga T. [Epidemiology of chronic subdural hematomas]. No Shinkei Geka. 2011;39(12):1149–53. 22128269

[11] Shimohigoshi W, Takase H, Iwashita H, Kawasaki T, Kobayashi Y, Takagi R, Higashijima T, Ozaki S, Fushimi S, Miyata Y, et al. PAR-1 expression in chronic subdural hematoma: potential association with vascular permeability. Neurotrauma Rep. 2025;6(1):956–62. 41141400 10.1177/2689288X251383714 PMC12549182

[12] Osuka K, Ohmichi Y, Ohmichi M, Nakura T, Iwami K, Watanabe Y, Miyachi S. Sequential expression of chemokines in chronic subdural hematoma fluids after trepanation surgery. J Neurotrauma. 2021;38(14):1979–87. 33497585 10.1089/neu.2020.7401

[13] Schievink WI, Maya MM, Moser FG, Tourje J. Spectrum of subdural fluid collections in spontaneous intracranial hypotension. J Neurosurg. 2005;103(4):608–13. 16266041 10.3171/jns.2005.103.4.0608

[14] Shimohigoshi W, Takase H, Haze T, Kobayashi Y, Manaka H, Kawasaki T, Sakata K, Yamamoto T. Renin-angiotensin-aldosterone system inhibitors as a risk factor for chronic subdural hematoma recurrence: A matter of debate. J Stroke Cerebrovasc Dis. 2023;32(10):107291. 37579641 10.1016/j.jstrokecerebrovasdis.2023.107291

[15] Page F. Intracranial hypotension. Lancet. 1953;1(6749):1–5. 13011928 10.1016/s0140-6736(53)92509-4

[16] Holmes JM. Intracranial hypotension associated with subdural haematoma. Br Med J. 1953;1(4824):1363–6. 13042259 10.1136/bmj.1.4824.1363 PMC2016656

[17] Hejazi N, Al-Witry M, Witzmann A. Bilateral subdural effusion and cerebral displacement associated with spontaneous intracranial hypotension: diagnostic and management strategies. Report of two cases. J Neurosurg. 2002;96(5):956–9. 12005407 10.3171/jns.2002.96.5.0956

[18] Schievink WI, Meyer FB, Atkinson JL, Mokri B. Spontaneous spinal cerebrospinal fluid leaks and intracranial hypotension. J Neurosurg. 1996;84(4):598–605. 8613851 10.3171/jns.1996.84.4.0598

[19] Ozyigit A. Spontaneous intracranial hypotension complicated by unilateral subdural hematoma, coma, and the rare kernohan’s Notch phenomenon. Clin Case Rep. 2023;11(1):e6899. 36703772 10.1002/ccr3.6899 PMC9871412

[20] Osada Y, Shibahara I, Nakagawa A, Sakata H, Niizuma K, Saito R, Kanamori M, Fujimura M, Suzuki S, Tominaga T. Unilateral chronic subdural hematoma due to spontaneous intracranial hypotension: a report of four cases. Br J Neurosurg. 2020;34(6):632–7. 31535558 10.1080/02688697.2019.1667482

[21] Collange O, Wolff V, Cebula H, Pradignac A, Meyer A, Kindo M, Diemunsch P, Proust F, Mertes PM, Kremer S. Spontaneous intracranial hypotension: an etiology for consciousness disorder and coma. Case Rep. 2016;7(10):207–11. 10.1213/XAA.0000000000000385 27552236

[22] Loya JJ, Mindea SA, Yu H, Venkatasubramanian C, Chang SD, Burns TC. Intracranial hypotension producing reversible coma: a systematic review, including three new cases. J Neurosurg. 2012;117(3):615–28. 22725982 10.3171/2012.4.JNS112030

[23] Takahashi K, Mima T, Akiba Y. Chronic subdural hematoma associated with spontaneous intracranial hypotension: therapeutic strategies and outcomes of 55 cases. Neurol Med Chir (Tokyo). 2016;56(2):69–76. 26489406 10.2176/nmc.oa.2015-0032 PMC4756246

[24] Hennig R, Kloster R. Burr hole evacuation of chronic subdural haematomas followed by continuous inflow and outflow irrigation. Acta Neurochir (Wien). 1999;141(2):171–6. 10189499 10.1007/s007010050282

[25] Lai T-H, Fuh J-L, Lirng J-F, Tsai P-H, Wang S-J. Subdural haematoma in patients with spontaneous intracranial hypotension. Cephalalgia. 2007;27(2):133–8. 17257233 10.1111/j.1468-2982.2006.01249.x

[26] Huang YH, Yang KY, Lee TC, Liao CC. Bilateral chronic subdural hematoma: what is the clinical significance? Int J Surg. 2013;11(7):544–8. 23707986 10.1016/j.ijsu.2013.05.007

[27] Oh JW, Kim SH, Whang K. Traumatic cerebrospinal fluid leak: diagnosis and management. Korean J Neurotrauma. 2017;13(2):63–7. 29201836 10.13004/kjnt.2017.13.2.63 PMC5702760

[28] Bossuyt PM, Reitsma JB, Bruns DE, Gatsonis CA, Glasziou PP, Irwig L, Lijmer JG, Moher D, Rennie D, de Vet HC, et al. STARD 2015: an updated list of essential items for reporting diagnostic accuracy studies. BMJ. 2015;351:h5527. 26511519 10.1136/bmj.h5527 PMC4623764

[29] Upadhyaya P, Ailani J. A review of spontaneous intracranial hypotension. Curr Neurol Neurosci Rep. 2019;19(5):22. 30888542 10.1007/s11910-019-0938-7

[30] Nishikawa M, Bolognese PA, Kula RW, Ikuno H, Takami T, Ohata K. Surgical management of Chiari malformations: preliminary results of surgery according to the mechanisms of ptosis of the brain stem and cerebellum. J Neurol Surg B Skull Base. 2021;82(2):264–72. 33816049 10.1055/s-0039-1697977 PMC8009696

[31] Nakaguchi H, Tanishima T, Yoshimasu N. Factors in the natural history of chronic subdural hematomas that influence their postoperative recurrence. J Neurosurg. 2001;95(2):256–62. 11780895 10.3171/jns.2001.95.2.0256

[32] Stanisic M, Pripp AH. A reliable grading system for prediction of chronic subdural hematoma recurrence requiring reoperation after initial Burr-Hole surgery. Neurosurgery. 2017;81(5):752–60. 28379528 10.1093/neuros/nyx090 PMC5808673

[33] Park HR, Lee KS, Shim JJ, Yoon SM, Bae HG, Doh JW. Multiple densities of the chronic subdural hematoma in CT scans. J Korean Neurosurg Soc. 2013;54(1):38–41. 24044079 10.3340/jkns.2013.54.1.38 PMC3772285

[34] Gharehbagh SS, Rasmussen BK, Smilkov E, Jensen RH. Spontaneous intracranial hypotension presenting with progressive cognitive decline. BMJ Case Rep. 2021;14(7). 10.1136/bcr-2020-241285 PMC8296796 34290004

[35] Hounsfield GN. Nobel award address. Computed medical imaging. Med Phys. 1980;7(4):283–90. 6993911 10.1118/1.594709

[36] Edlmann E, Giorgi-Coll S, Whitfield PC, Carpenter KLH, Hutchinson PJ. Pathophysiology of chronic subdural haematoma: inflammation, angiogenesis and implications for pharmacotherapy. J Neuroinflammation. 2017;14(1):108. 28558815 10.1186/s12974-017-0881-y PMC5450087

[37] Toi H, Kinoshita K, Hirai S, Takai H, Hara K, Matsushita N, Matsubara S, Otani M, Muramatsu K, Matsuda S, et al. Present epidemiology of chronic subdural hematoma in japan: analysis of 63,358 cases recorded in a National administrative database. J Neurosurg. 2018;128(1):222–8. 28156246 10.3171/2016.9.JNS16623

[38] Rajbhandari S, Gurung P, Nepal G, Acharya S, Pant B. A case of postpartum chronic subdural hematoma. Clin Case Rep. 2021;9(8):e04694. 34457300 10.1002/ccr3.4694 PMC8380432

[39] Chandankhede AR, Thombre SD. When a headache means more: A case report of acute spontaneous subdural hematoma after spinal anesthesia for caesarean section. Cureus. 2023;15(4):e37917. 37220432 10.7759/cureus.37917 PMC10200024

[40] Luetzen N, Dovi-Akue P, Fung C, Beck J, Urbach H. Spontaneous intracranial hypotension: diagnostic and therapeutic workup. Neuroradiology. 2021;63(11):1765–72. 34297176 10.1007/s00234-021-02766-z PMC8528761

[41] Rauhala M, Helen P, Seppa K, Huhtala H, Iverson GL, Niskakangas T, Ohman J, Luoto TM. Long-term excess mortality after chronic subdural hematoma. Acta Neurochir (Wien). 2020;162(6):1467–78. 32146525 10.1007/s00701-020-04278-w PMC7235063

[42] Mea E, Chiapparini L, Savoiardo M, Franzini A, Bussone G, Leone M. Headache attributed to spontaneous intracranial hypotension. Neurol Sci. 2008;29(Suppl 1):S164–165. 18545924 10.1007/s10072-008-0914-5

[43] Terakado T, Omi A, Matsumaru Y, Ishikawa E. Two cases of chronic subdural hematoma with spontaneous intracranial hypotention treated with hematoma drainage followed by epidural blood patch under intracranial pressure monitoring. NMC Case Rep J. 2023;10:93–8. 37131497 10.2176/jns-nmc.2022-0356 PMC10149143

[44] Matsumura A, Anno I, Kimura H, Ishikawa E, Nose T. Diagnosis of spontaneous intracranial hypotension by using magnetic resonance myelography. Case report. J Neurosurg. 2000;92(5):873–6. 10794305 10.3171/jns.2000.92.5.0873

[45] Schievink WI. Spontaneous intracranial hypotension. N Engl J Med. 2021;385(23):2173–8. 34874632 10.1056/NEJMra2101561

[46] Dobrocky T, Grunder L, Breiding PS, Branca M, Limacher A, Mosimann PJ, Mordasini P, Zibold F, Haeni L, Jesse CM, et al. Assessing spinal cerebrospinal fluid leaks in spontaneous intracranial hypotension with a scoring system based on brain magnetic resonance imaging findings. JAMA Neurol. 2019;76(5):580–7. 30776059 10.1001/jamaneurol.2018.4921 PMC6515981

[47] Ferrante E, Regna-Gladin C, Arpino I, Rubino F, Porrinis L, Ferrante MM, Citterio A. Pseudo-subarachnoid hemorrhage: a potential imaging pitfall associated with spontaneous intracranial hypotension. Clin Neurol Neurosurg. 2013;115(11):2324–8. 24075686 10.1016/j.clineuro.2013.08.028

[48] Iijima J, Hoshi K, Ito H, Kanno M, Murakami Y, Takahashi K, Matsumoto K, Yamaguchi Y, Nakajima M, Miyajima M, et al. Total transferrin in cerebrospinal fluid is a novel biomarker for spontaneous intracranial hypotension. Fukushima J Med Sci. 2021;67(2):64–70. 34373399 10.5387/fms.2020-19 PMC8460282

[49] Takase H, Tatezuki J, Ikegaya N, Yamamoto D, Hashimoto M, Takagi M, Mochimatsu Y, Kawahara N. Therapeutic suggestions for chronic subdural hematoma associated with idiopathic thrombocytopenic purpura: A case report and literature review. NMC Case Rep J. 2015;2(3):118–22. 28663980 10.2176/nmccrj.2014-0209 PMC5364897

[50] Murakami M, Morikawa K, Matsuno A, Kaneda K, Nagashima T. Spontaneous intracranial hypotension associated with bilateral chronic subdural hematomas–case report. Neurol Med Chir (Tokyo). 2000;40(9):484–8. 11021083 10.2176/nmc.40.484

[51] Nakajima H, Sakai T, Aoki N, Takakura K. Bilateral chronic subdural hematomas associated with intracranial hypotension–case report. Neurol Med Chir (Tokyo). 1996;36(9):647–9. 8913082 10.2176/nmc.36.647

[52] Hsieh CT, Su IC, Hsu SK, Huang CT, Lian FJ, Chang CJ. Chronic subdural hematoma: differences between unilateral and bilateral occurrence. J Clin Neurosci. 2016;34:252–8. 27742369 10.1016/j.jocn.2016.09.015

[53] Callen AL, Dillon WP, Shah VN. Unusual neuroimaging findings in spontaneous intracranial hypotension. Neuroradiology. 2023;65(5):875–82. 36879063 10.1007/s00234-023-03136-7

[54] Callen AL, Fakhri M, Timpone VM, Thaker AA, Dillon WP, Shah VN. Temporal characteristics of CSF venous fistulas on dynamic decubitus CT myelography: a retrospective Multi-Institution cohort study. AJNR Am J Neuroradiol. 2023. 10.3174/ajnr.A8078 PMC10756577 38123910

[55] Beck J, Gralla J, Fung C, Ulrich CT, Schucht P, Fichtner J, Andereggen L, Gosau M, Hattingen E, Gutbrod K, et al. Spinal cerebrospinal fluid leak as the cause of chronic subdural hematomas in nongeriatric patients. J Neurosurg. 2014;121(6):1380–7. 25036203 10.3171/2014.6.JNS14550

[56] Honda M, Maeda H. Intraoperative hematoma volume can predict chronic subdural hematoma recurrence. Surg Neurol Int. 2021;12:232. 34221563 10.25259/SNI_97_2021 PMC8247721

[57] Karakaya Z, SariTas A, Yesim Akyol P, Esad Topal F, Payza U, Bilgİn S. Evaluation of chronic subdural hematoma volume calculated via cavalieri’s principle. Konuralp Med J. 2019;11(2):260–8.

[58] Sulioti G, Gray L, Amrhein TJ. Popping the balloon: abrupt onset of a spinal CSF leak and spontaneous intracranial hypotension in idiopathic intracranial hypertension, a case report. Headache. 2022;62(2):208–11. 35072949 10.1111/head.14264

[59] Madhavan AA, Carr CM, Benson JC, Brinjikji W, Diehn FE, Kim DK, Lehman VT, Liebo GB, Morris PP, Shlapak DP, et al. Diagnostic yield of intrathecal gadolinium MR myelography for CSF leak localization. Clin Neuroradiol. 2022;32(2):537–45. 34292360 10.1007/s00062-021-01060-y

[60] Kaur S, Kwon K, Ramachandran S, Pisinski L, Krauthamer A. A case of spontaneous intracranial hypotension in a 45-year-old male with headache, behavior changes and altered mental status. Radiol Case Rep. 2022;17(7):2289–94. 35570871 10.1016/j.radcr.2022.03.075 PMC9092074

