# Supplementary material for: Parasagittal subdural space: a novel quantitative marker of spontaneous intracranial hypotension syndrome-induced chronic subdural hematoma
Source: BMC Med Imaging. 2025 Dec 29;25:514. doi: 10.1186/s12880-025-02065-6 (PMC12751203; doi:10.1186/s12880-025-02065-6)
Supplement: Supplementary file 1 — Supplementary Material 1 [file 12880_2025_2065_MOESM1_ESM.docx]

# **SUPPLEMENTARY MATERIALS**

**Parasagittal subdural space: A novel quantitative marker of spontaneous intracranial hypotension syndrome-induced chronic subdural hematoma**

Takahiro Tanaka, MD, PhD^1#^, Hajime Takase, MD, PhD ^2, 3#*^, Tatsuya Haze, MD, PhD ^3, 4, 5^, Wataru Shimohigoshi, MD ^1^, Mitsuru Sato, MD, PhD ^1^, Tetsuya Yamamoto, MD, PhD ^1^

^#^These two authors equally contributed to this work.

^1^ Department of Neurosurgery, Yokohama City University, Yokohama, Kanagawa, Japan

^2^ Departments of Radiology and Neurology, Massachusetts General Hospital and Harvard Medical School, Charlestown, MA, USA.

^3^ YCU Center for Novel and Exploratory Clinical Trials (Y-NEXT), Yokohama City University Hospital, Yokohama, Japan.

^4^ Department of Medical Science and Cardiorenal Medicine, Yokohama City University Graduate School of Medicine, Yokohama, Japan.

^5^ Department of Nephrology and Hypertension, Yokohama City University Medical Center, Yokohama, Japan

*Correspondence: Hajime Takase, MD, PhD.

Departments of Radiology and Neurology, Massachusetts General Hospital and Harvard Medical School, 149 13^th^ St., Charlestown, MA 02129, USA.

E-mail: [HTAKASE@mgh.harvard.edu](mailto:HTAKASE@mgh.harvard.edu)

**Results**

**Table S1.** Characteristics of the cohort of non-spontaneous intracranial hypotension syndrome (SIH) bilateral CSDH. Univariate analyses for the difference between the subgroups bilateral symmetric and asymmetric chronic subdural hematoma (CSDH). Mann-Whitney U test, Fisher’s exact- or Chi-squared test as appropriate. CI, confidence interval. OR, odds ratio. BMI, body mass index. IHF, interhemispheric fissure.

**
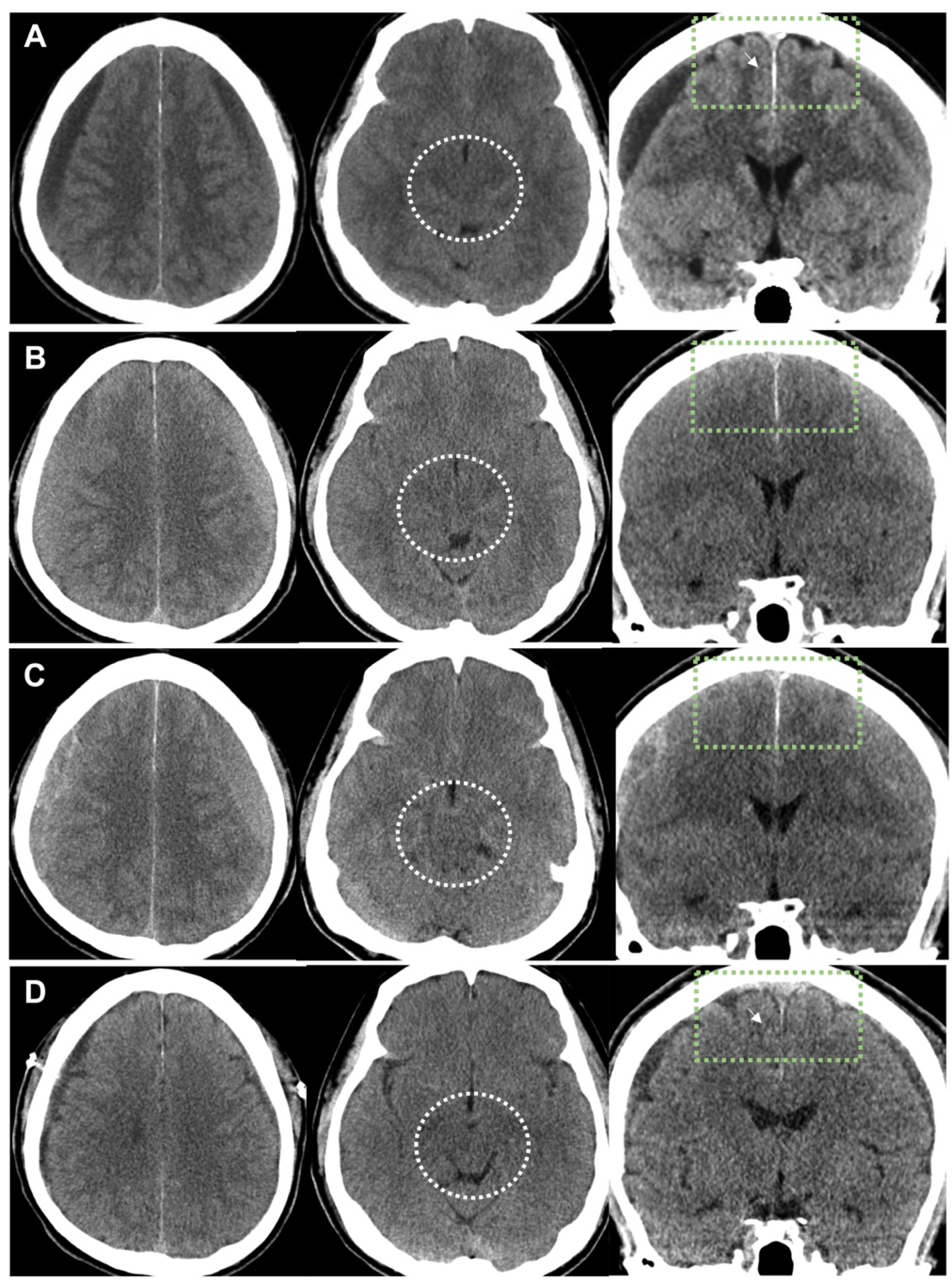
**

**Figure S1.** Time series of head CT of a case of bilateral CSDH with SIH (Case 8: 54-year-old man with headache).

(**A: Initial**) (Left) symmetric bilateral CSDH, (Center) obliteration of crural-and-ambient cisterns (white dotted circle), (Right) preserved PSS (green dotted rectangle) and cerebral sulci in the IHF (white arrow head), suggesting SIH-induced bilateral CSDH.

(**B: Right after EBP, headache improved**) As conservative therapy failed and hematoma density became iso-high, EBP was performed. (Center) the cisterns remained obliterated (white dotted circle). (Right) tightened PSS and obliterated cerebral sulci in the IHF (green dotted rectangle), suggesting growing non-SIH-related CSDH and/or resolution of SIH.

(**C: 1 week after EBP: before ID**) headache deteriorated. (Left) grown hematoma, (Center) slightly improved obliterated cisterns (white dotted circle), (Right) tightened PSS and obliterated cerebral sulci in the IHF (green dotted rectangle), suggesting resolution of SIH and growing non-SIH-related CSDH.

(**D: 1 week after ID**) all clinical symptoms improved after ID. (Center) preserved cisterns (white dotted circle), (Right) preserved PSS (green dotted rectangle) and cerebral sulci in the IHF (white arrow head), indicating that SIH-induced bilateral CSDH was successfully treated.

SIH: spontaneous intracranial hypotension syndrome, CSDH: chronic subdural hematoma, PSS: parasagittal subdural space, EBP: epidural blood patch, ID: irrigation and drainage.
