# Supplementary figures and images for: Parasagittal subdural space: a novel quantitative marker of spontaneous intracranial hypotension syndrome-induced chronic subdural hematoma
Source: BMC Med Imaging. 2025 Dec 29;25:514. doi: 10.1186/s12880-025-02065-6 (PMC12751203; doi:10.1186/s12880-025-02065-6)

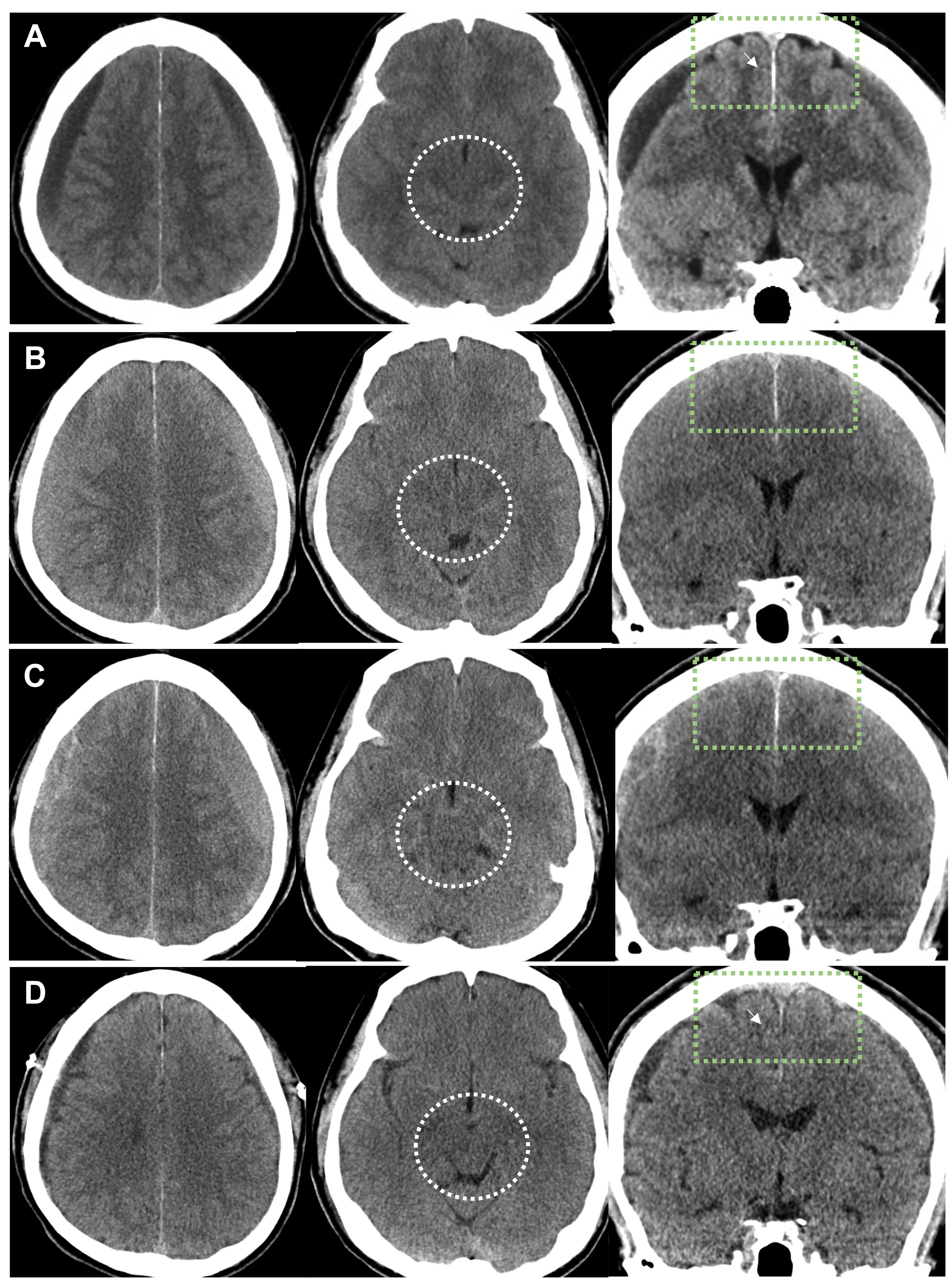

Supplement: Supplementary file 3 — Supplementary Material 3 [file 12880_2025_2065_MOESM3_ESM.jpg]
